# Psychophysical Evaluation of a Sanshool Derivative (Alkylamide) and the Elucidation of Mechanisms Subserving Tingle

**DOI:** 10.1371/journal.pone.0009520

**Published:** 2010-03-03

**Authors:** Kelly C. Albin, Christopher T. Simons

**Affiliations:** 1 Givaudan Flavors Corporation, Cincinnati, Ohio, United States of America; 2 School of Medicine, University of California San Diego, San Diego, California, United States of America; Duke University, United States of America

## Abstract

Previous studies investigated the neural and molecular underpinnings of the tingle sensation evoked by sanshool and other natural or synthetic alkylamides. Currently, we sought to characterize the psychophysical properties associated with administration of these compounds. Like other chemesthetic stimuli, the synthetic tingle analog isobutylalkylamide (IBA) evoked a sensation that was temporally dynamic. Repeated IBA application at short (30 sec) interstimulus intervals (ISI) resulted in a tingle sensation that increased across trials. Application at longer ISIs (∼30 min) resulted in a sensation of decreased intensity consistent with self-desensitization. Prior treatment with the TRPV1 or TRPA1 agonists, capsaicin and mustard oil did not cross-desensitize the tingle sensation evoked by IBA suggesting that neither TRPV1 nor TRPA1 participate in the transduction mechanism sub-serving tingle. When evaluated over 30-min time period, lingual IBA evoked a sensation that was described initially as tingling and pungent but after approximately 15 min, as a cooling sensation. Further, we found that the sensation evoked by lingual IBA was potentiated by simultaneous application of cold (0°C) and cool (21°C) thermal stimuli but was unaffected by warm (33°C) and hot (41°C) temperatures. Finally, to test the hypothesis that the tingling sensation is subserved by the activation of mechanosensitve fibers, we evaluated lingual tactile thresholds in the presence and absence of lingual IBA. The presence of IBA significantly raised lingual tactile thresholds, whereas capsaicin did not, identifying a role for mechanosensitive fibers in conveying the tingle sensation evoked by sanshool-like compounds. Collectively, these results show that lingual alkylamide evokes a complex sensation that is temporally dynamic and consistent with *in vitro* and *in vivo* experiments suggesting these compounds activate mechanosensitve neurons via blockade of KCNK two-pore potassium channels to induce the novel tingling sensation.

## Introduction

Alkylamides are a unique class of compounds that elicit a distinctive tingling sensation when applied to mucosal surfaces [1]. Two natural alkylamides, α-hydroxy-sanshool and spilanthol are found in Szechuan pepper (Xanthoxylum piperitum) and Jambu fruit (Acmella oleracea), respectively and are used in ethnic cuisines to provide unique oral sensations during the consumption of meals [2], [3]. Medicinal uses of these compounds have also been described; the plants have been used indigenously as analgesics, digestive aids and are purported to stimulate immune responses [4]. The unique tingling sensation evoked by these compounds is qualitatively different from the pungent sensations evoked by other natural products [1] including capsaicin, thiocyanates, and cinnamic aldehyde, which have been shown to activate TRP receptors expressed in the terminals of peripheral nociceptive fibers [5]–[7]. Previous studies have sought to define the neural and molecular underpinnings of the tingle sensation. *In vivo*, electrophysiological studies have shown that α-hydroxy-sanshool activates low and high threshold cold-sensitive fibers as well as low threshold mechanosensitive fibers of the rat lingual nerve [1]. Consistent with these findings, we recently reported that when injected into the rat hindpaw, a stable derivative of α-hydroxy-sanshool (isobutylalkenyl amide; IBA) activated both wide-dynamic range (WDR) and low-threshold mechanoreceptors (LTM) in the spinal dorsal horn [8]. *In vitro,* α-hydroxy-sanshool has been shown to activate two types of sensory cells: nociceptive neurons expressing TRPV1 but not TRPA1 (although see [9], [10]) and large diameter, TrkC-expressing, mechanosensitive neurons [11]. Initial studies on transduction mechanisms purported that α-hydroxy-sanshool-evoked activity in nociceptive cells occurred through activation of TRPV1 [9], [12] and TRPA1 [9]. Subsequent reports suggest that activation of both nociceptive and mechanosensitve cells occurs through the unique ability of alkylamides to inhibit background potassium conductances through anesthetic-sensitive two-pore potassium channels (KCNK3, KCNK9 and KCNK18; [11]).

Despite the recent flurry of studies investigating the physiological mechanisms underlying tingling, little work has examined the psychophysical properties of the alkylamides. Initial work showed that the sensation elicited by oral application of α-hydroxy-sanshool was qualitatively different from the burning sensation typically evoked by TRPV1 agonists [1]. Subsequent studies determined the threshold concentration and duration of sensation for a variety of naturally occurring sanshools [12]. No studies, to our knowledge, have evaluated the temporal aspects of repeated alkylamide application. Such studies provide unique insights into the molecular and neural mechanisms subserving these sensations. For instance, chemoirritants display unique temporal effects such as sensitization and desensitization (for review see [13]). Sensitization occurs when capsaicin (or other irritant compounds including piperine, menthol, cinnamic aldehyde) is applied to mucosal surfaces with relatively short (<2 min) interstimulus intervals (ISI) and the intensity of the perceived irritation builds with each application [14]–[19]. This phenomenon has been proposed to result from either an increase in excitability of peripheral and/or central nociceptive neurons or from spatial summation [13]. Desensitization, on the other hand, occurs at relatively long ISIs (>5 min), is characterized by a reduction of the perceived intensity of subsequent chemoirritant applications [17], [20]–[22] and has been shown to have neural and molecular underpinnings. Specifically, both nociceptive neurons [23] and TRP receptors [24] show reduced responsivity (tachyphylaxis) to repeated application of irritant chemicals that is calcium-dependent [25], [26]. Currently, we sought to determine if alkylamides, like other chemesthetic compounds, evoke sensitizing and/or desensitizing patterns of sensation that are ISI dependent. Such a finding would be unique because to date, sensitization and desensitization have only been described for compounds that evoke pungent sensations through activation of nociceptive neurons. Similarly, we investigated if the tingling sensation evoked by alkylamides could be cross-desensitized by specific TRPV1 or TRPA1 agonists. Results from these studies would shed light on the neural mechanisms subserving tingling. Finally, *in vivo* studies suggest that α-hydroxy-sanshool activates cold-sensitive fibers whereas *in vitro* studies suggest that α-hydroxy-sanshool activates nociceptive and mechanosensitive neurons. As such, we sought to characterize whether lingual alkylamide application evoked distinct tingling, pungent or cold sensations that were temporally defined. We further hypothesized that if alkylamides evoke a cool sensation it should be potentiated or reduced by the simultaneous application of cold or warm temperatures, respectively. Similarly, we hypothesized if the tingling sensation is subserved by the activation of mechanosensitve fibers, application of alkylamide compounds should modulate tactile thresholds.

## Materials and Methods

### General Procedures

#### Subjects

A total of 68 subjects ranging in age from 20–59 participated in these experiments and were recruited via phone solicitation. Subjects were asked to refrain from smoking or eating spicy food for a minimum of 2 hours prior to any experiment as chemoirritants can induce desensitization that, in some cases, can last longer than 1 hour. Subjects were allowed to participate in more than 1 experiment. Panelists were paid for their participation in each experiment. All studies were approved by the Givaudan Flavors Corp. Institutional Review Board (IRB) and participants gave written informed consent. Givaudan's IRB functions under the governing principles of Title 45 in the Code of Federal Regulations (CFR) Part 46, and includes adherence to the FDA's requirements outlining the composition of an IRB (21 CFR 56.107). As such, the IRB consists of both Givaudan employees and non-employees as well as scientific and lay persons. The Board was set-up, trained and is advised by another non-Givaudan employee to further ensure the committee operates with the participants best interests in mind.

#### Chemicals

**Figure 1:**
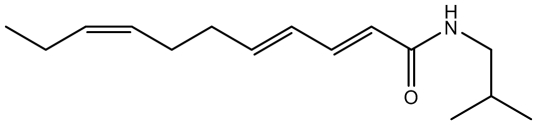
Chemical structure of isobutylalkylamide (IBA) used to evoke tingle sensation. Compound was synthesized according to (27).

N-isobutyl (2E, 4E, 8Z)-unadeca-2,4,8-trienamide (IBA), a proprietary synthetic stable analog ([27]; 98% purity), was obtained from Givaudan commercial flavor stocks (Cincinnati, OH) and solubilized in polyethylene glycol (PG) to a final concentration of 0.52%. IBA is structurally very similar to α-hydroxy-sanshool but is more stable due to the lack of extended conjugation (figure 1). The increased stability of this compound provides advantages over other naturally occurring alkylamides, such as sanshool and spilanthol, which oxidize relatively quickly under aqueous and natural lighting conditions. Degradation of these active compounds results in lower activity and increased likelihood of off-target effects. Similarly, stock solutions of the TRPV1 and TRPA1 agonists, capsaicin (Sigma-Aldrich, St. Louis MO) and mustard oil (allyl isothiocyante; Acros Organics, NJ) were made. Capsaicin was initially dissolved in a 95% ethanol solution to a concentration of 0.1% and then diluted with distilled water to a final concentration of 10 ppm. Mustard oil was used at a concentration of 0.125% by solubilizing in PG. Capsaicin and mustard oil concentrations were selected because they have previously been used to evoke significant self- and cross-desensitization [28].

### Specific Procedures

#### Experiment 1: Self-sensitization/desensitization

Twenty-five subjects (11 male, 14 female) ranging in age from 20–58 participated in this experiment. Prior to the actual collection of data, each panelist participated in a rehearsal session during which they practiced placing 10 ml aliquots of water into one half of their mouth and subsequently expectorating, while simultaneously minimizing solution contact with the opposite side of the mouth. On the day of the experiment, panelists were given 10 ml of the IBA in a plastic cup (Solo, NJ) and instructed to place the entire volume into one side of their mouth. The side of the mouth receiving treatment was counterbalanced across subjects. After 15 seconds, subjects expectorated the solution and 5 seconds later rated the perceived tingle intensity using the gLMS [29]. Ten seconds later, subjects placed another 10 ml sample into the same side of their mouth, held the sample for 15 seconds, expectorated and rated the perceived intensity again. This process occurred every 30 seconds (see figure S1) until 5 ratings were obtained. Following the last rating, subjects rinsed their mouth with distilled water and waited quietly for a minimum of 30 min until the tingle sensation disappeared. Following the rest period, the presence of desensitization was assessed. Panelists were asked to take 20 ml of the IBA solution into their mouth, swirl the contents throughout the entire oral cavity for 15 secs and expectorate. Subjects subsequently performed a 2-alternative forced-choice (2-AFC) procedure and chose the side of their mouth having the strongest tingling sensation. They also provided bilateral intensity ratings using the gLMS.

#### Data analysis

As data obtained from the gLMS is typically distributed log-normally across subjects [29], the ratings data were normalized by converting to log_10_ prior to statistical analysis. The presence of sensitization was assessed by subjecting the five initial ratings data to a repeated-measures analysis of variance (ANOVA) with post-hoc Tukey's HSD tests; panelist and application were main effects. The presence of desensitization was assessed by using a binomial analysis to establish whether a significant majority of subjects chose the previously untreated side of the mouth as having a stronger tingling sensation following a 30 min hiatus. Similarly, significant differences in the mean tingle ratings on both sides of the mouth were assessed using a Bonferroni corrected t-test. All data are presented as mean ± SE and an α-level of 0.05 was taken as significant.

#### Experiment 2: Cross-desensitization

Thirty subjects (19 male, 11 female) participated in the capsaicin experiment whereas 30 subjects (9 male, 21 female) participated at a later time in the mustard oil experiments. All subjects were between the age of 23 and 59 years of age. In order to evaluate the effect of capsaicin or mustard oil desensitization on tingle perception, we used the half-tongue, two-alternative forced–choice procedure reported previously (e.g. [28]). We chose specifically to evaluate IBA-evoked tingle as opposed to pungency, because we were interested whether this sensation is mediated by a population of fibers separate from those sensitive to capsaicin or mustard oil. Capsaicin or mustard oil (∼40 µL) was applied unilaterally to one half of the dorsal lingual surface using a cotton-tipped applicator. On the contralateral side, a control solution containing 0.95% ethanol (for capsaicin control) or PG (for mustard oil control) was simultaneously applied in a similar manner. The side of the tongue receiving capsaicin or mustard oil was counterbalanced across subjects. Subjects were then asked to rest their tongue quietly on the floor of the mouth for 10 min or until the pungent sensation disappeared, whichever came last. Following the rest period, two 1-cm diameter filter paper disks (Whatman International LTD., Maidstone, UK) were each saturated with 20 µL of IBA and placed bilaterally onto the tongue surface which had previously been treated with capsaicin or mustard oil (or control solution). After approximately 10–15 sec, subjects were asked to select the side of the tongue having the strongest tingling sensation and also to provide bilateral tingle intensity ratings using a 0–10 intensity scale (0 = no sensation 10 = strongest sensation imaginable). A 0–10 intensity scale was used in this experiment due to logistical constraints associated with using paper versions of the gLMS. However, the main advantage of the gLMS is for use in across-subject studies where differences in scale use can potentially confound results [29]. As our studies were within-subject designs, differences in scale use are less important and we do not believe that the use of the 0–10 intensity scale compromised the reliability of our results.

#### Data analysis

A binomial analysis was used to assess whether a significant majority of subjects chose the previously untreated side of the tongue as having a stronger tingling sensation following capsaicin or mustard oil pretreatment. Significant differences in the mean tingle ratings on the capsaicin-treated (or mustard oil treated) and untreated sides of the tongue were assessed using a Bonferroni corrected t-test. All data are presented as mean ± SE and an α-level of 0.05 was taken as significant.

#### Experiment 3: Time intensity

A modified time intensity procedure was used to assess whether IBA can evoke tingling, pungent or cold sensations that are temporally distinct. Twenty-eight subjects (10 male, 18 female) participated in this study. Cotton-tipped applicators were used to paint ca. 40 µL of IBA onto the anterior dorsal surface of the tongue. Every 60 sec for 32 min, subjects were asked to rate the overall perceived intensity using a 0–10 intensity scale (0 = no sensation 10 = strongest sensation imaginable) and then select the predominant sensation(s) experienced at that time point from a list of descriptors; subjects were not restricted in the number of attributes they could select. The list of descriptors was comprised of common somatosensory terms and included “tingling/pricking, anesthetized/numb, cooling, warming, and burning/irritation/pungent”. In an effort to ensure that all participants had the same definition for each attribute, a verbal description of each was given prior to the study's initiation. In a separate control experiment, subjects used the same modified time intensity methodology to rate the perceived intensity of attributes associated with lingual application of vehicle (PG).

#### Data analysis

The mean overall intensity at each time point was calculated across all subjects to construct a composite curve. Maximum intensity (Imax) and time to Imax (Tmax) were extracted from the composite curve. At all time points, the proportion of subjects selecting each attribute was determined. Calculated values were then plotted for each attribute.

#### Experiment 4: Thermal-tingle interactions

Twenty-nine panelists (11 males, 18 females) aged 23–51 were used to study the influence of temperature on the IBA-evoked sensation. IBA (∼40 µL) was painted onto the anterior dorsal surface of one side of the tongue using a cotton-tipped applicator. A control solution of PG was similarly applied to the contralateral side. The side receiving IBA was counterbalanced across subjects. After 1 min, subjects were asked to attend to the difference in sensation across the two sides of their tongue; this difference was referred to as d_o_. Subjects were then given 1 of 4 cups (Solo, NJ) containing distilled water held at the following temperatures: 0°C, 21°C, 33°C, 41°C. Solutions were pre-poured into the cups and brought to temperature by placing into thermally-controlled baths. The solutions were placed in front of the subject in random order and each panelist was asked to place the sample in their mouth. Subjects were asked to compare the difference in tingle sensation across the two sides of their tongue in the absence of the thermal stimulus (d_o_) and in the presence of the thermal stimulus (d_1_; see figure S2). They were then asked to rate the intensity of d_1_ relative to d_o_ using a 9-point bipolar scale as shown in figure S2. Panelists performed the same task for each of the 4 thermal solutions and the entire process lasted approximately 5 minutes.

#### Data analysis

The categorical data were transformed to numerical data ranging from -4 (d_1_ extremely less intense compared to d_0_) to +4 (d_1_ extremely more intense compared to d_0_) with the condition “d_1_ same intensity as d_0_” receiving a value of 0. As the nature of the data were still categorical, the effect of temperature on the tingle sensation was assessed using Friedman's test (adjusted for ties) with Wilcoxon, Nemenyi, McDonald-Thompson post-hoc tests [30]; panelist and temperature were main effects. An α-level of 0.05 was taken as significant.

#### Experiment 5: Tingle-tactile interactions

Thirty-one subjects (16 males, 15 females) ranging in age from 25–56 years of age participated in this experiment. Lingual tactile sensitivity was tested by applying a von Frey filament (Stoelting, Chicago, IL) calibrated to 0.008 or 0.02 N to the anterior third of the tongue. In some conditions, no stimulus was applied (blank). Each condition was tested, in randomized order, 10 times for a total of 30 trials. Subjects were blindfolded and responded by indicating whether or not they felt the stimulus and whether or not they were sure of their response. From these data, response matrices for each subject were constructed from which indices representing the tactile sensitivity of the tongue were calculated (R-index; [31]). This procedure has been used previously to assess lingual tactile sensitivity following dorzolamide treatment [32]. IBA (∼40 µL) was then applied to the dorsal surface of the anterior tongue using a cotton-tipped applicator. After approximately 1 min, tactile sensitivity was tested again, using exactly the same procedure indicated previously. Finally, in a control experiment, the effect of the nociceptive stimulus capsaicin (10 ppm) on tactile sensitivity was assessed. Thirty panelists (15 males, 15 females) participated in a pre-stimulus assessment followed immediately by a post-capsaicin assessment. Panelists used the same procedure as indicated above.

#### Data analysis

Panelist sensitivity to the two tactile stimuli was assessed by calculating the R-index [31]. The R-index estimates the classical signal detection measure of sensitivity P(A) - the area under a receiver operating characteristic (ROC) curve [31]. The area under an ROC curve, and hence the R-index, ranges from 0.5 (chance level discrimination) to 1 (perfect discrimination) and measures an individual's ability to discriminate between two stimuli, in this case the presence or absence of a tactile stimulus. The effect of IBA on tactile sensitivity was assessed using ANOVA and a binomial analysis was used to determine whether a significant majority of panelists were able to detect the stimuli under each treatment condition. All data are presented as means ± SE and an α-level of 0.05 was taken as significant.

## Results

### Experiment 1: Self-Sensitization/Desensitization

**Figure 2:**
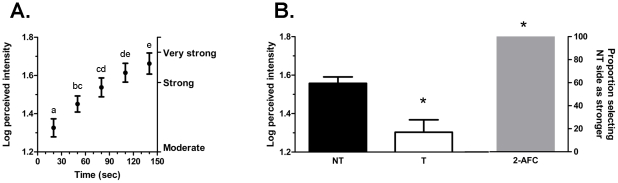
Temporal characteristics of tingle sensation evoked by repeated application of IBA. (A) Repeated application of IBA at 30-sec intervals evoked a tingle sensation that grew progressively in intensity. The intensity of the tingle sensation evoked by each application was greater than that evoked by preceding applications. Error bars indicate SEM and letters above bars indicate significant differences at p<0.05. (B) Panel shows a set of bar graphs. The left-hand pair plots the intensity ratings of tingle sensation for the non-treated (NT, black bar) and previously treated (T, open bar) side of the tongue, respectively. The gray bar to the right indicates the proportion of subjects choosing the non-treated side as yielding a stronger tingle sensation in the 2-AFC test. Error bars indicate SEM. * above open bar indicates significant difference (p<0.05) between pretreated and nontreated sides of the tongue. * above gray bar indicates significant majority (p<0.05) of subjects chose non-treated side as having stronger tingling sensation.

IBA (0.52%) evoked an initial tingling sensation that was perceived, on average, as being moderately intense (figure 2a). Consistent with sensitization, the repeated application of IBA (30 sec ISI) evoked a tingling sensation that grew significantly (F_4,96_ = 26.7; p<0.001) in intensity (figure 2a). Indeed, the second application was found to elicit a tingle that was significantly (p = 0.018) more intense than the initial application. With each subsequent application, perceived intensity continued to increase until the fifth application which was not perceived as significantly more intense than the fourth application.

Results from 2-AFC testing confirm the presence of self-desensitization. Following a 30 min hiatus, a significant majority of subjects (24/25; p<0.001) chose the previously untreated side of the tongue as having a stronger tingle sensation when IBA was applied bilaterally (figure 2b, right hand bar). Consistent with this finding, the mean tingling intensity of the untreated-side of the tongue (1.58±0.02) was found to be significantly (p<0.001) higher than the previously treated side (1.29±0.06; figure 2b, left hand bars).

### Experiment 2: Cross-Desensitization

**Figure 3:**
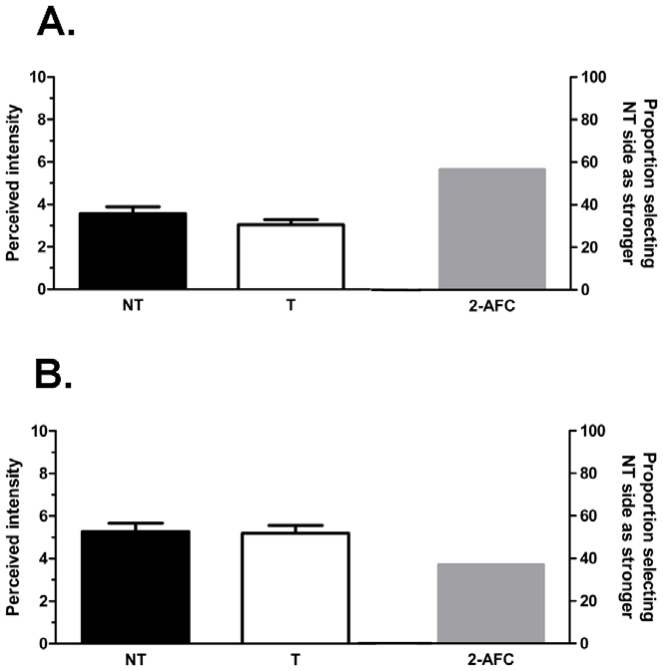
Lack of cross-desensitization of IBA-evoked tingle. Desensitization was assessed following lingual pre-treatment with (A) capsaicin (10 ppm) or (B) mustard oil (0.125%). Both panels show the bilateral intensity ratings and 2-AFC data when tested after the capsaicin (A) or mustard oil (B) burn had subsided. The format is the same as in figure 2.

Prior application of capsaicin or mustard oil had no effect on the perceived tingle sensation evoked by lingual IBA. Desensitization was evoked by applying capsaicin or mustard oil to the lingual surface and waiting for a minimum of 10 minutes for the irritant sensation to dissipate; prior to testing with IBA, panelists confirmed that the pungent sensation had disappeared. Following the bilateral application of IBA, a non-significant (p = 0.585) majority of subjects (17/30) chose the side not previously receiving capsaicin pre-treatment as having a stronger tingling sensation (figure 3a, right hand bar). Consistent with this finding, no significant (p = 0.104) difference in mean tingle intensity was found between the capsaicin-treated and untreated sides (3.04±0.25 vs 3.57±0.32, respectively; figure 3a, left hand bars). A similar finding was observed with mustard oil. A non-significant (p = 0.200) majority of subjects (19/30) chose the side not previously receiving mustard oil as having the stronger tingling sensation (figure 3b, right hand bar) and no significant differences (p = 0.845) were observed for the mean tingling ratings assigned to the treated (5.18±0.39) and untreated (5.28±0.39) sides of the tongue (figure 3b, left hand bar).

### Experiment 3: Time Intensity

**Figure 4:**
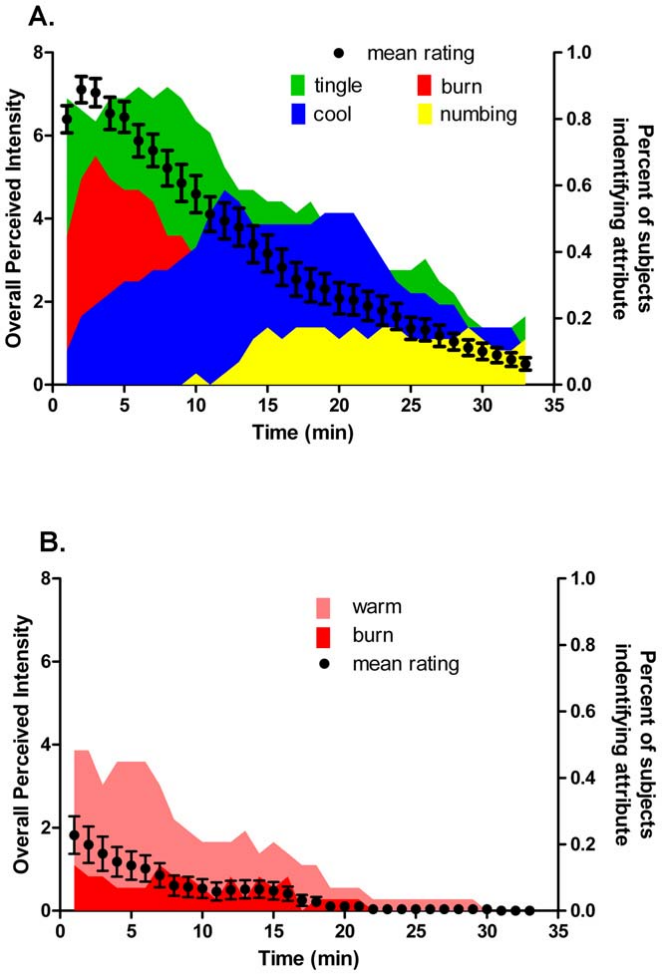
Temporal profile and attribute identification following lingual IBA (or vehicle) application. (A) Figure shows the mean overall intensity perceived over time (black dots) and the proportion of panelists selecting tingle (green), burning (red), cooling (blue) or numbing (yellow) at each time point. Note that initially, lingual IBA evoked a sensation that was characterized as both tingling and burning followed later by a sensation described primarily as tingling and cooling with some evidence of anesthesia. Error bars indicate SEM. (B) Application of vehicle (PG) elicited neither tingle nor cooling but did evoke an initial sensation characterized as warming. Format as in A.

The application of IBA to the lingual surface elicited a sensation that was temporally dynamic (figure 4a black circles). IBA evoked a tingling sensation with a maximum intensity of 7.11±0.32 that occurred 1 min after administration. Thereafter, the overall intensity of the sensation slowly decayed and by 30 minutes, most subjects reported an absence of any sensation. Interestingly, IBA evoked a sensation that displayed temporally distinct sensory qualities (figure 4a colored areas). Over the first 9 min, the sensation was described primarily as tingling but also having a significant burning quality. Warming, cooling and anesthetized were used very infrequently during this time period. After *ca.* 9 min, burning was no longer a dominant attribute and instead, the sensation was described as having tingling and cooling properties. Tingling was perceived as the dominant attribute compared to cooling until *ca*. 19 min, after which the sensation was described as predominantly cooling with some tingling. Although used infrequently, some subjects did identify a numbing sensation occurring approximately 10 min after lingual administration. These data suggest that IBA, in addition to the expected tingling, pungent and anesthetic sensations, evokes a cooling sensation that is of long duration and relatively low intensity. The control application of PG elicited a very different response profile compared to IBA and was described primarily as warming over the ∼10 min duration during which a sensation was identified (figure 4b.)

### Experiment 4: Thermal-Tingle Interactions

**Figure 5:**
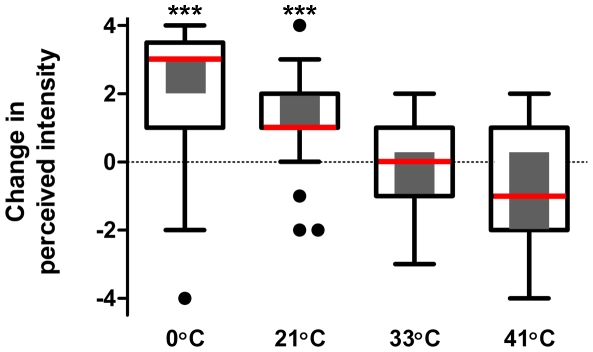
Effect of temperature on the perceived sensation evoked by lingual IBA. Cold (0°C) and cool (21°C) temperatures potentiated the sensation evoked following lingual IBA whereas warm (37°C) and hot (41°C) temperatures had no effect. Box-whisker plots demonstrate the distribution of responses obtained from individuals at each temperature. White boxes on box-whisker plots correspond to the inter-quartile range (25%–75%), vertical lines indicate range of responses, the red horizontal line indicates median values and the black circles indicate outlier responses as determined by Tukey's analysis. The grey bar inside each white box indicates the 95% confidence interval. Confidence intervals not crossing the abscissa indicate that perceived intensity was significantly (p<0.05) different from 0 (no difference between d_0_ and d_1_; see Figure S2 for scale) and are denoted by the *** above the appropriate box-whisker plot.

Data from experiment 3 suggested that, in addition to a tingling sensation, IBA evoked a cooling sensation that became notable approximately 12 minutes after lingual application. As such, we hypothesized that the IBA-evoked cooling sensation should be enhanced by the presence of a cold thermal stimulus and reduced in the presence of a warm thermal stimulus. The results from the current study suggest lingual IBA potentiates the sensation evoked by cold or cool temperatures but is unaffected by warm or hot temperatures. The difference in perceived intensity between the IBA-treated and non-treated sides of the tongue was significantly (X^2^ = 41.3; p<0.001) affected by temperature (figure 5). Post-hoc tests revealed that the perceived intensity difference was greatest (2.50) in the presence of a cold (0°C) stimulus followed by a cool, room temperature (21°C) stimulus (1.25). The warm (37°C) and hot (41°C) stimuli did not significantly alter the intensity difference (−0.25 vs. −0.50, respectively) between d_o_ and d_1_. Non-parametric one-sample sign tests revealed that at low temperatures (0°C and 21°C), the perceived intensity difference between d_o_ and d_1_ was significantly (both tests p<0.001) greater than 0 indicating that the low temperatures potentiated the sensation evoked by IBA (figure 5). In contrast, the perceived intensity differences between d_0_ and d_1_ when warm and hot temperatures were applied were not significantly different (p = 0.664 and p = 0.308, respectively) from 0 indicating that higher temperatures had no effect on the IBA-evoked sensation (figure 5).

### Experiment 5: Tingle-Tactile Interactions

**Figure 6:**
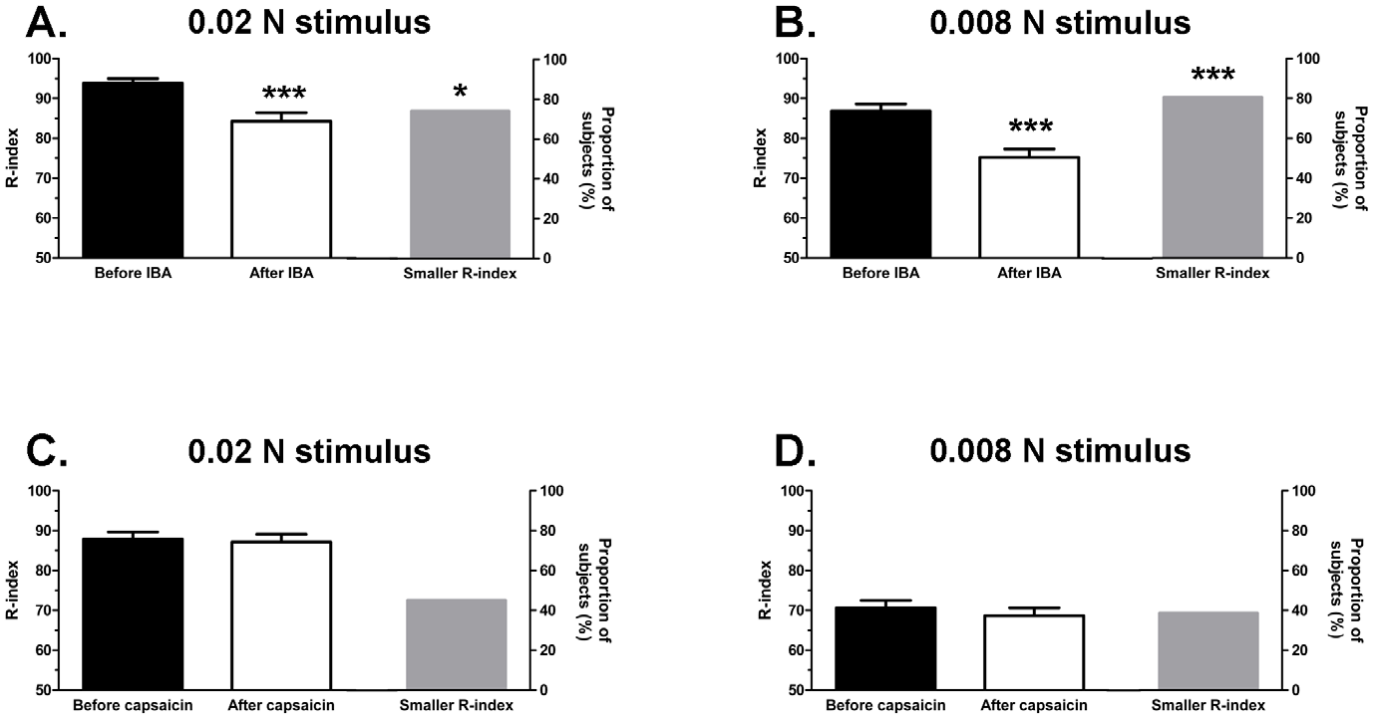
Effect of IBA and capsaicin on lingual tactile sensitivity. Each panel shows a pair of bar braphs. The left-hand pair plots the average R-index before (black bar) and immediately after (open bar) lingual IBA (or capsaicin) application. R-index is a derived measure of sensitivity ranging from 50–100%. A value of 50% represents the chance level of detection for a subject to distinguish between signal (tactile stimulus) and noise (no tactile stimulus) whereas a value of 100% indicates perfect discrimination. Error bars indicate SEM. * above open bar indicates significant differences in tactile sensitivity following lingual IBA (or capsaicin) administration. The grey bar to the right of each panel represents the proportion of subjects that had a smaller R-index (indicating worse detection) following lingual IBA (or capsaicin) administration. * above grey bar indicates a significant proportion of subjects had a smaller R-index following lingual IBA (or capsaicin). (A) Sensitivity to a 0.02N tactile stimulus was significantly reduced following lingual IBA (0.52%). (B) Sensitivity to a 0.008N tactile stimulus was significantly reduced following lingual IBA (0.52%) administration. (C) Sensitivity to a 0.02N tactile stimulus was not affected following lingual capsaicin (10 ppm) administration. (D) Sensitivity to a 0.008N tactile stimulus was not affected following lingual capsaicin (10 ppm).

Alkylamides are purported to activiate mechanosensitive neurons [1], [11] and we hypothesized that the presence of IBA on the anterior dorsal surface of the tongue would result in decreased lingual tactile sensitivity. Prior to IBA application, all subjects were able to detect the 0.02 N stimulus and a significant (p<0.001) majority of subjects (28 of 31) were able to detect the 0.008 N stimulus. Across all panelists, the average R-index was 93.8±1.2 and 86.9±1.7 for the 0.02 N and 0.008 N stimuli, respectively (figure 6a and b). Following IBA application, during the period of active tingling, tactile sensitivity was decreased. The average R-index was significantly lower after IBA for both the 0.02 N (84.3±2.1; p<0.001) and 0.008 N (75.2±2.1; p<0.001) stimuli. Indeed, 6 of 31 subjects were unable to detect the 0.02 N stimulus and 11 of 31 subjects were unable to detect the 0.008 N stimulus. A significant majority of subjects scored lower on the tactile sensitivity test following IBA (figure 6a and b) for the 0.02 N (23 of 31; p = 0.031) and 0.008 N (25 of 31; p<0.001) tests.

To control for a generalized attentional effect as well as the fact that IBA activates nociceptive neurons, tactile sensitivity was assessed, in a separate group of subjects, following lingual capsaicin application. Prior to capsaicin application, a significant (p<0.001) majority of subjects (31 of 31) detected the 0.02 N stimulus whereas a non-significant (p = 0.471) majority of subjects (18 of 31) detected the 0.008 N stimulus. Interestingly, the pre-capsaicin R-indices are lower for both the 0.02 N and 0.008 N stimuli as compared to the pre-IBA R-indices. These differences likely reflect simple group differences since different subjects were used in each test. In contrast to lingual IBA application, capsaicin did not significantly alter lingual tactile sensitivity. There was no significant difference between the average R-indices obtained before or after capsaicin application (figure 6c and d) for either the 0.02 N (87.9±1.8 vs. 87.2±1.9, respectively; p = 0.456) or 0.008 N (70.6±1.9 vs. 68.7±1.9; p = 0.687) stimuli. Moreover, whereas the presence of IBA affected the tactile sensitivity of a significant majority of subjects, capsaicin did not. Only 14 of 31 subjects (p = 0.762) had a lower R-index in the 0.02 N condition and 12 of 31 subjects (p = 0.925) in the 0.008N condition (figure 6c and d).

## Discussion

Lingual application of alkylamides elicits a unique sensation reported as tingling. Like other well-characterized chemesthetic agents [14]–[22], repeated application of IBA resulted in both sensitizing and desensitizing patterns of sensation and the evoked pattern was shown to be dependent upon the interstimulus interval. However, whereas most irritants can be cross-desensitized by prior application of TRPV1 or TRPA1 agonists, IBA was not. These results indicate that the tingle sensation (but not pungency) elicited by this compound is likely evoked through activity in a population of somatosensory neurons not expressing TRPV1 or TRPA1. Prior reports indicate that alkylamides activate tactile, nociceptive and thermally-sensitive neurons [1], [8]–[11]. Temporal profiling of the sensation evoked by lingual IBA application showed that all three attributes were routinely selected to characterize the sensation. Whereas tingling was identified as present throughout the 30 min testing period, pungency and cooling were noted only at the beginning and end, respectively. Consistent with our hypothesis that alkylamides have both thermal and tactile qualities, unique interactions were found when additional thermal or tactile stimuli were co-applied, although these findings may be specific for IBA. A cold stimulus was found to intensify the sensation evoked by lingual IBA whereas warm and hot stimuli were found to have no effect. Similarly, consistent with the proposed mechanism of alkylamides activating mechanosensitive neurons [1], [11], lingual tactile sensitivity was diminished in the presence of IBA but not capsaicin.

### Self-Sensitization/Desensitization

With repeated application, most chemesthetic stimuli evoke a sensitizing and/or desensitizing pattern of irritation as assessed psychophysically [14]–[22], [33], [34]. Similar findings have been reported in electrophysiology studies. *In vitro*, repeated applications of capsaicin [24] or nicotine [35] elicit tachyphylaxis in TRPV1 or nAChR expressing neurons, respectively. Similarly, recordings of neural activity in nociceptive-specific and wide dynamic range cells in the trigeminal caudalis (Vc) show patterns of increasing activity (sensitization) when capsaicin was delivered at short ISIs (<1 min) and decreasing activity (desensitization) when delivered at longer ISIs (>5 min; [23]). Similar findings in Vc were found with nicotine [23], menthol [36], mustard oil [37], NaCl [38], [39], and acid [38], [39]. Sensitization has been proposed, at least in part, to be due to spatial summation and the recruitment of previously quiescent neurons as the compound diffuses through lingual epithelium [13]. The mechanism underlying desensitization has not yet been fully elucidated, however, it appears to be calcium dependent [25], [26], [40]. Given that alkylamides have been shown to activate nociceptive pathways [1], [8]–[11], it perhaps is not surprising that repeated application of IBA evokes self-sensitization and desensitization. However, the predominant sensation evoked by the alkylamides is not one of pungency *per se*, but instead of robust tingling. It has recently been proposed that this tingling sensation is not due to activation of traditional irritant transduction mechanisms such as TRPA1 or TRPV1 [10]. Instead, the tingling is thought to be due to the blockade of two-pore potassium channels located on mechanosensitive neurons [11]. To our knowledge, the present findings are the first to report the sensitizing and desensitizing properties of compounds that activate mechanosensitive pathways. The mechanisms sub-serving sensitization and desensitization, however, may be independent of neuronal type. For instance, consistent with a proposed mechanism of capsaicin sensitization [13], IBA sensitization might also be due to spatial recruitment. Similarly, it would be interesting to determine *in vitro,* if IBA desensitization is also calcium dependent. Other mechanisms are also possible. The self-desensitizing effect could be a result of “halo-dumping” [41] in which the higher ratings on the untreated side of the mouth could have reflected scores in which panelists combined ratings for tingle and burning sensations. Alternatively, the KCNK two-pore potassium channels identified as being central to eliciting tingle [11] have also been implicated as having a role in anesthesia [11], [42].

### Cross-Desensitization

Unlike the pungency evoked by most chemoirritants, the tingle sensation elicited by IBA was not cross-desensitized by prior application of capsaicin or mustard oil. Studies of cross-desensitization have been used in prior investigations to obtain evidence of underlying neurobiological mechanisms [18], [19], [21], [28]. For instance, capsaicin cross-desensitization of citric acid [18], NaCl [19], menthol [43], and mustard oil [28] suggests that a common population of capsaicin-sensitive trigeminal nociceptive fibers convey sensations of oral irritation [43]. Similarly, the lack of cross-desensitization (e.g. between capsaicin and nicotine; [20]) has been taken as evidence that neural processing of some chemoirritants remains, at least partially, separate. In the present study, we saw no indication that prior treatment of lingual epithelium with capsaicin or mustard oil cross-desensitized the tingle sensation elicited by IBA. These results suggest that the molecular mechanism subserving the tingle sensation are TRPV1 and TRPA1 independent and that the tingle sensation is conveyed from the periphery to higher brain centers via a population of neurons that are neither capsaicin- nor mustard oil-sensitive. As capsaicin and mustard oil evoke activity in nociceptive fibers, IBA and other alkylamides are likely to evoke tingling through non-nociceptive, mechanosensitive pathways. This hypothesis is consistent with reports that sanshool activates mechanosensitive neurons via blockade of potassium leak channels [11].

### Time Intensity

Reports that alkylamides activate TRP channels involved in nociception [9]–[11] and afferent nociceptive fibers [1], [8] as well as anecdotal evidence that sanshool evokes sensations of pungency and cooling as well as tingle, prompted us to develop a methodology that would allow us to study the potentially dynamic sensation evoked by lingual IBA. Time intensity studies have been used extensively in the sensory sciences to study the temporal aspects of a given sensation [44]. However, this methodology only allows a single sensation to be tracked over time [44]. More recently, temporal dominance of sensation (TDS) has been developed in which panelists are asked to select and rate the intensity of the sensation that is dominant at any given time point [45]. The method has proven to be valuable in understanding the temporality of complex taste and flavor sensations (e.g. [46], [47]) Unfortunately, TDS requires significant panelist training and until recently, the technique required sophisticated self-built software for data collection. Taking inspiration from TDS we used a hybrid method in which panelists were given a list of potential descriptors and every minute over the course of 30 minutes, asked to select the attribute from the list that best represented the sensation perceived at that time point and rate the intensity. Consistent with the physiological reports, lingual application of IBA evoked a dynamic sensation that was characterized initially as primarily tingling with some burning, followed by primarily tingling with some cooling and lastly primarily cooling with some tingling. The term warming was rarely used over the 30-min time course indicating that IBA (and likely other alkylamides) does not initiate activity in warm-sensitive pathways via TRPV3 or TRPV4 activation. Interestingly, numbing was also selected, although relatively infrequently. Anecdotal reports suggest that alkylamides evoke a numbing sensation [1] and the KCNK channels targeted by these chemicals have been previously shown to be sensitive to volatile anesthetics [42]. As the subjects participating in this test were not extensively trained nor provided with attribute references or standards, it is possible that confusion over descriptor definition prevented them from selecting certain terms. Along similar lines, pricking is a term often used to denote painful sensations mediated by nociceptors. We made the effort to align subjects on attribute definitions by providing verbal descriptors of each prior to the initiation of the study. Specifically, we told subjects that the tingle/pricking attribute was a buzzing sensation akin to that evoked when putting the tongue on the terminals of a 9-volt battery and not necessarily painful. However, it is still possible that confusion over the definition of this attribute resulted in tingle/pricking being selected when in fact subject's intended to identify a descriptor having sharp, painful qualities. However, despite these limitations, we were able to document that lingual IBA evokes a complex sensation that changes over time. The dominant tingling sensation is likely due to blockade of two-pore potassium channels [10], [11] expressed by lingual mechanoreceptors, whereas the burning sensation evoked at early time intervals is likely due to activation of TRPV1- [8]–[11] or TRPA1- [9], [10] expressing nociceptors. Although in early reports [1] it was noted that sanshool activated cold-sensitive fibers in the rat lingual nerve, we report the first evidence that alkylamides can evoke a cooling sensation in humans. The cooling was not identified as a predominant component of the overall sensation evoked by lingual IBA until *ca*. 15 minutes post-application after which time it increased in prevalence. The delayed onset may reflect certain pharmacokinetics associated with IBA or its cognate receptor. Alternatively, it may reflect the fact that the tingling and/or burning sensations masked the cooling component at early time points and did not become noticeable until these other sensations had decreased in magnitude. Similar findings have been observed with mixtures of other chemesthetic chemicals. For instance, capsaicin-evoked irritation is less intense in the presence of menthol than when delivered at the same concentration as a single stimulus [48]. Using this same logic, it is possible that IBA-evoked cooling mitigated the burning and/or tingling component at earlier times as well.

### Thermal-Tingle Interactions

Initial physiological studies suggested that sanshool elicited activity in cold-sensitive lingual fibers [1]. Our time intensity data confirmed the presence of an IBA-evoked cooling sensation but it had a significantly delayed onset and was of relatively weak intensity (see above). To further explore the relationship between temperature and IBA-evoked sensations, we assessed the impact of tingling on various thermal sensations. We hypothesized that if IBA evoked a cooling sensation in addition to tingle, the overall intensity should be enhanced by the presence of a cold thermal stimulus and reduced in the presence of a warm thermal stimulus. Prior studies have shown that application of menthol increases the perceived intensity of a cold stimulus and reduces the perceived intensity of a warm stimulus [49], [50]. Similarly, the burning sensation evoked by capsaicin is exacerbated by administration of a moderately warm to hot stimulus and mitigated by co-application of a cold stimulus [51]. Results of our study did confirm the presence of thermal-tingle interactions. Addition of a cold (0°C) or cool (21°C) stimulus significantly enhanced the overall IBA-evoked sensation whereas the addition of a warm (37°C) or hot (41°C) stimulus had no effect. That the warm stimulus had no effect was anticipated because we chose a temperature that was equivalent to that seen in the oral cavity and it was expected to be a neutral stimulus. However, we did expect the hot stimulus to significantly reduce the perceived intensity of the IBA-evoked sensation because we anticipated that it would neutralize the IBA-cooling component. Although the difference was not significant, application of the hot stimulus tended to reduce the perceived intensity following lingual IBA. It is possible that a thermal stimulus of higher temperature would have decreased the cooling component even further; however lingual application was not possible because of the potential for tissue damage. Finally, it is of great interest to speculate on the receptor mechanism subserving the cooling sensation associated with lingual alkylamide delivery. Prior studies have identified some two-pore potassium channels as being thermally sensitive [52], [53] with progressive channel closing (and depolarization) at cold temperatures. However, as yet it is not known whether the specific channels sensitive to alkylamides (KCNK3, KCNK9, and KCNK12) are also thermally sensitive. *In vitro*, IBA nor any of the naturally occurring sanshool derivatives evoked activity in transfected or stable cell lines expressing TRPM8 (unpublished results). This suggests that the IBA-sensitive KCNK channels might also be co-expressed in cold-sensitive neurons expressing TRPM8 and blockade of these two-pore potassium channels by alkylamides would evoke a cold sensation. However, to date, KCNK channels have not been reported in these cell types. Alternatively, TRPA1 has been suggested to be involved in the transmission of cold pain [54] and it is possible that this receptor contributes to the cold sensation identified herein. Finally, it is possible that a, as yet, unidentified receptor is responsible for the cooling sensation evoked by IBA. Further studies are needed to delineate which of these mechanisms are responsible.

### Tingle-Tactile Interactions

Utilizing sensitive Signal Detection methodology [31], [55], we assessed the impact of lingual IBA and capsaicin administration on tactile thresholds. Signal Detection theory provides a framework in which sensory psychologists can test subject's ability to distinguish sensory “signals”from background noise [31], [55]. In this regard, we tested the ability of our subjects to identify a tactile “signal”in the absence and presence of IBA- or capsaicin-evoked “noise”. We reasoned that if alkylamides activated mechanosensitive pathways thereby increasing background “noise”, tactile thresholds would be increased. On the other hand, if alkylamides activated nociceptive pathways via TRPV1 or TRPA1, the effect of IBA on tactile thresholds should be similar to that seen with capsaicin. We found that lingual IBA had a dramatic effect on tactile thresholds making it difficult for subjects to identify the presence of a tactile stimulus. This effect was likely separate from any that may have occurred due to alkylamide's ability to evoke a numbing sensation, as tactile sensitivity was assessed within the first 5 min following lingual administration. As observed in the time intensity studies, tingle was evoked immediately following lingual IBA administration whereas the presence of anesthesia was noted infrequently and generally not until *ca*. 10 min had elapsed. A reduction in tactile sensitivity was not seen following capsaicin administration. Taken together, these results are consistent with the hypothesis that alkylamides elicit a tingling sensation via activation of mechanosensitive pathways. In contrast, capsaicin evokes activity in nociceptive pathways and, as suggested by our results, appears to be processed separately from tactile information. It is of interest that capsaicin had no effect on tactile sensitivity. TRPV1 plays an important role in inflammatory hyperalgesia and allodynia and intradermal capsaicin injection has been used extensively as a model of inflammatory pain (for review see [56]). We anticipated that following lingual capsaicin administration, primary and/or secondary hyperalgesia and allodynia would develop causing subjects to be more sensitive to a tactile stimulus. Similar effects on tactile sensitivity have been observed following intradermal injection into human hairy skin [57], [58]. Primary hyperalgesia occurs within the area of tissue injury and has been attributed to peripheral release of inflammatory mediators that increase the sensitivity of primary nociceptive fibers to noxious stimuli (for review see [59]). On the other hand, secondary hyperalgesia and allodynia results in increased sensitivity to noxious and non-noxious stimuli outside of the area of tissue injury and involves the central sensitization of spinothalamic tract neurons via a mechanism that depends on the activation of several second messenger cascades (PKC, PKA, NO/PKG, etc.) involved in excitatory and inhibitory signal transduction pathways [60]. It is possible that the dose of capsaicin used in the present study was too low to induce significant inflammation. Alternatively, as capsaicin is most often encountered in food, mechanisms within the oral cavity may prevent or minimize the inflammatory response.

In summary, the present results show that alkylamides evoke a dynamic, multidimensional sensation that can sensitize or desensitize with repeated application. However, unlike the sensation evoked by other known chemesthetic agents, alkylamide-evoked tingle is unlikely due to activity within nociceptive pathways. Instead, the data presented herein are consistent with *in vitro* and *in vivo* experiments that suggest alkylamides activate mechanosensitve neurons via blockade of KCNK two-pore potassium channels to induce the novel tingling sensation.

## Supporting Information

Figure S1 Experimental design used to test sensitization of IBA-evoked tingle. IBA was taken into one side of the oral cavity every 30 sec. Fifteen seconds after the oral exposure of IBA, the compound was expectorated and 5 sec later, panelists rated the perceived intensity using the gLMS (29).
(0.56 MB TIF)

Figure S2 Procedure and scale used to assess the impact of thermal stimuli on the sensation evoked by IBA. One half of the tongue was painted with IBA and the sensory difference between the treated and untreated sides of the tongue is referred to as d_0_. Subjects are then asked to attend to the sensory difference (d_1_) when a thermal stimulus is co-applied (e.g., 0°C water). For each thermal stimulus, subjects were asked to compare d_0_ and d_1_ and select the point on the scale that best reflected their sensory experience.
(0.66 MB TIF)
