# Supplementary figures and images for: Psychophysical Evaluation of a Sanshool Derivative (Alkylamide) and the Elucidation of Mechanisms Subserving Tingle
Source: PLoS One. 2010 Mar 3;5(3):e9520. doi: 10.1371/journal.pone.0009520 (PMC2831077; doi:10.1371/journal.pone.0009520)

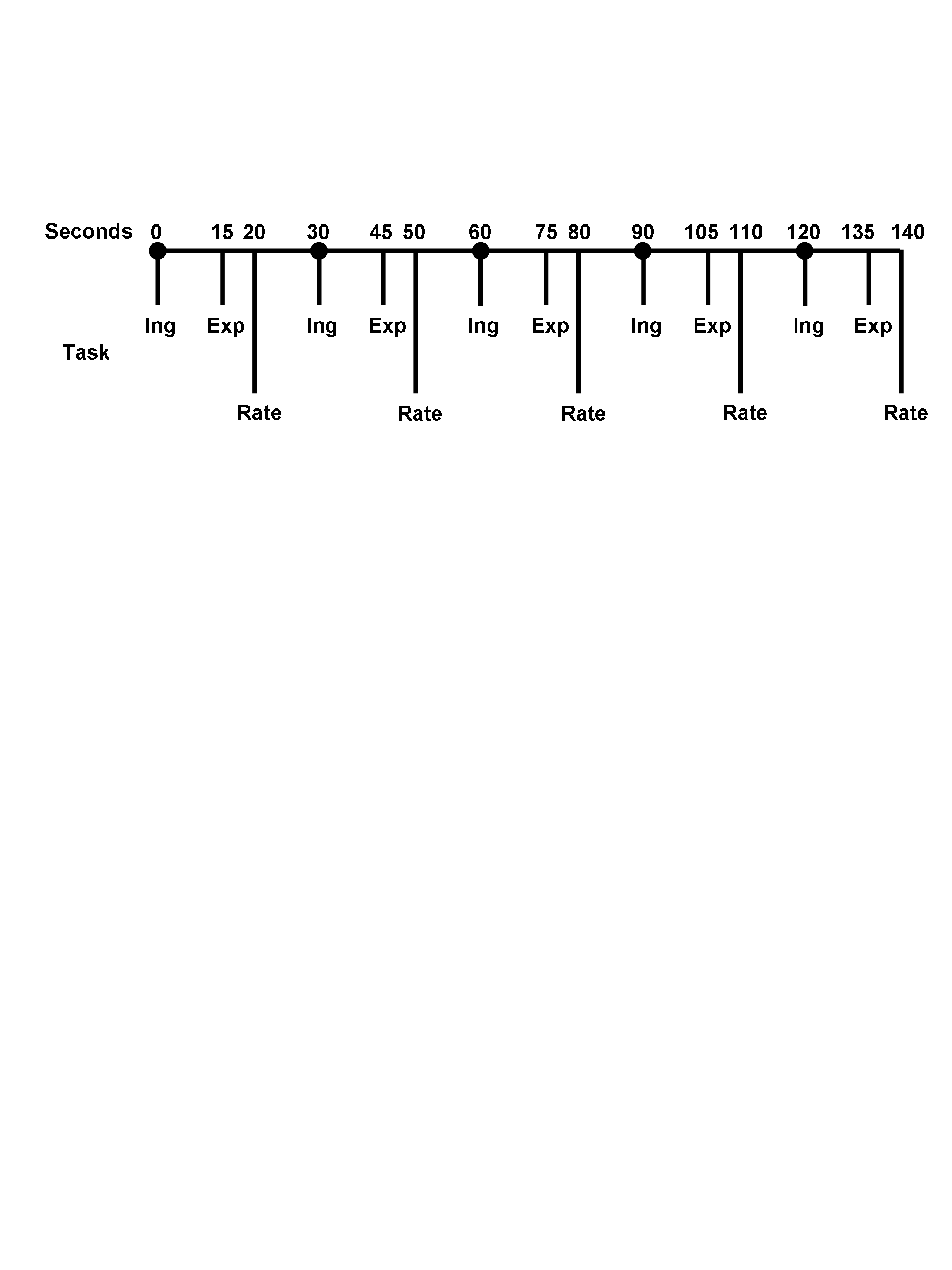

Supplement: Figure S1 — Experimental design used to test sensitization of IBA-evoked tingle. IBA was taken into one side of the oral cavity every 30 sec. Fifteen seconds after the oral exposure of IBA, the compound was expectorated and 5 sec later, panelists rated the perceived intensity using the gLMS (29). (0.56 MB TIF) [file pone.0009520.s001.tif]

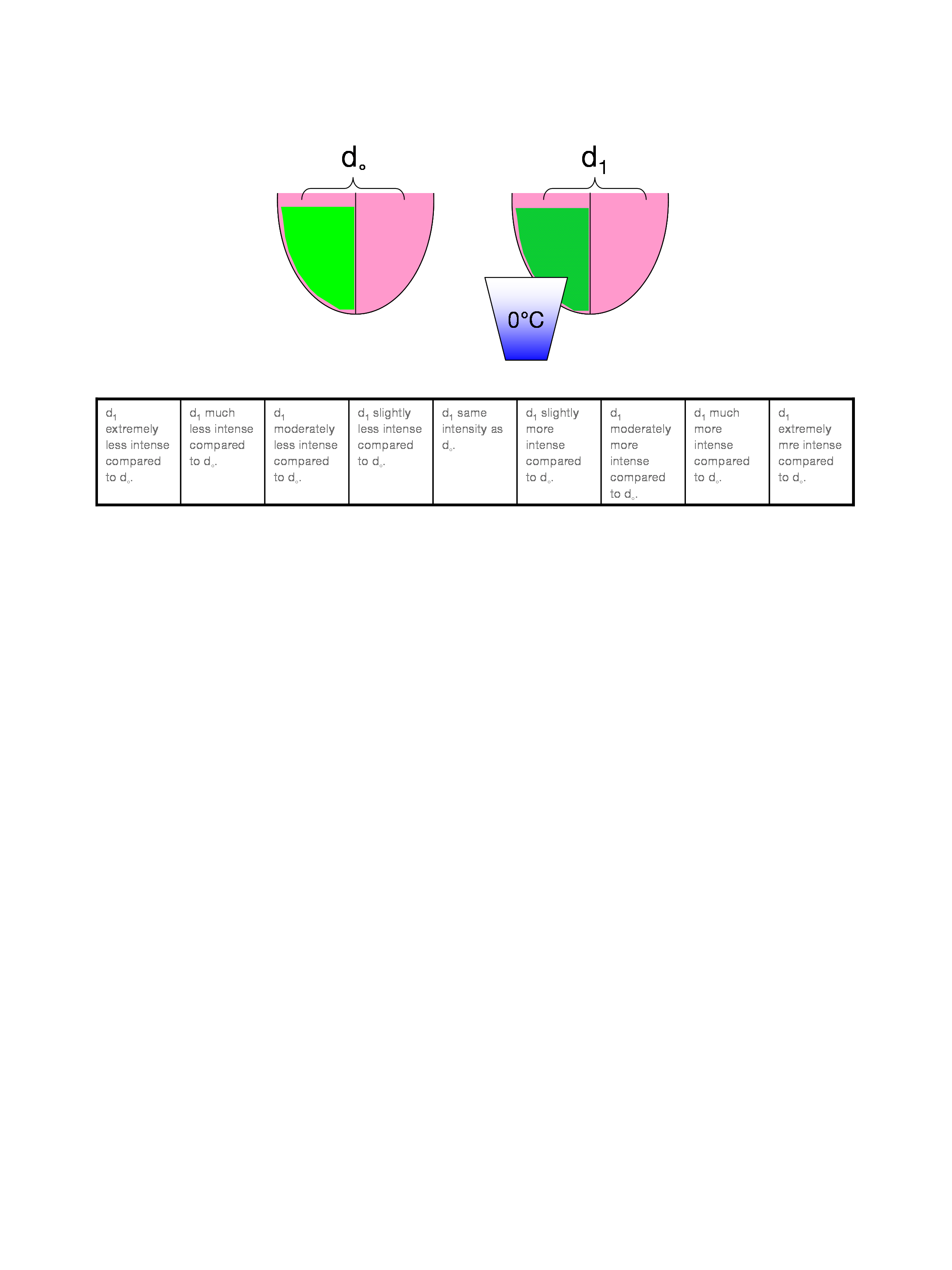

Supplement: Figure S2 — Procedure and scale used to assess the impact of thermal stimuli on the sensation evoked by IBA. One half of the tongue was painted with IBA and the sensory difference between the treated and untreated sides of the tongue is referred to as d0. Subjects are then asked to attend to the sensory difference (d1) when a thermal stimulus is co-applied (e.g., 0°C water). For each thermal stimulus, subjects were asked to compare d0 and d1 and select the point on the scale that best reflected their sensory experience. (0.66 MB TIF) [file pone.0009520.s002.tif]
